# Wafer-Level 3D Integration Based on Poly (Diallyl Phthalate) Adhesive Bonding

**DOI:** 10.3390/mi12121586

**Published:** 2021-12-20

**Authors:** Zhong Fang, Peng You, Yijie Jia, Xuchao Pan, Yunlei Shi, Junjie Jiao, Yong He

**Affiliations:** 1 School of Mechanical Engineering, Nanjing University of Science and Technology, Nanjing 210094, China; fangzhongah@163.com (Z.F.); youpeng@njust.edu.cn (P.Y.); pxchxc@njust.edu.cn (X.P.); jjj120@njust.edu.cn (J.J.); 2 No. 208 Research Institute of China Ordnance Industries, Beijing 102202, China; jy-j@163.com; 3 Quality Inspection and Testing Center, China Electronic Product Reliability and Environmental Testing Research Institute, Guangzhou 510610, China; 13770562272@163.com

**Keywords:** 3D integration, adhesive bonding, poly (diallyl phthalate), SOI wafer, wafer thinning

## Abstract

Three-dimensional integration technology provides a promising total solution that can be used to achieve system-level integration with high function density and low cost. In this study, a wafer-level 3D integration technology using PDAP as an intermediate bonding polymer was applied effectively for integration with an SOI wafer and dummy a CMOS wafer. The influences of the procedure parameters on the adhesive bonding effects were determined by Si–Glass adhesive bonding tests. It was found that the bonding pressure, pre-curing conditions, spin coating conditions, and cleanliness have a significant influence on the bonding results. The optimal procedure parameters for PDAP adhesive bonding were obtained through analysis and comparison. The 3D integration tests were conducted according to these optimal parameters. In the tests, process optimization was focused on Si handle-layer etching, PDAP layer etching, and Au pillar electroplating. After that, the optimal process conditions for the 3D integration process were achieved. The 3D integration applications of the micro-bolometer array and the micro-bridge resistor array were presented. It was confirmed that 3D integration based on PDAP adhesive bonding is suitable for the fabrication of system-on-chip when using MEMS and IC integration and that it is especially useful for the fabrication of low-cost suspended-microstructure on-CMOS-chip systems.

## 1. Introduction

The last few decades have seen an astonishing increase in the functionality and complexity of microsystems [1,2]. This tendency has been driven by the development of 3D integration technology. By stacking microelectromechanical units or integrated circuit units on top of each other and using vertical interconnections between the units, micro-systems can achieve high levels of function and system integration. In addition, micro-systems with 3D integration technology have the advantages of short interconnection circuits, small parasitic capacitance, and inductance [3,4,5,6]. This technology allows membranes or microstructures to be directly fabricated on the handle wafer and for integrated circuits to be fabricated on another wafer, respectively; after that, the wafers are bonded together and are interconnected by 3D integration.

The key to 3D integration is low temperature wafer-level bonding, such as plasma-enhanced direct bonding, anodic bonding, thermos-compression bonding, adhesive bonding, etc. [7,8,9]. Compared to other bonding technologies, adhesive bonding offers several advantages: (a) the bonding temperature is usually below 350 centigrade and has good compatibility with the CMOS process; (b) it is suitable for a wide variety of bonding interfaces does not have any special requirements; (c) the surface topography can be fully covered by a bonding polymer; and (d) the whole process is simple and is inexpensive [9,10]. Due to these advantages of adhesive bonding, 3D integration that is based on adhesive bonding has been a research hotspot for many years. Recently, various micro-system applications have been reported for this kind of 3D integration, such as micro-bolometer arrays [11,12], movable micro-mirror arrays [13,14], and radio frequency micro-systems [15].

The most commonly used adhesive polymers in the bonding process are thermosetting epoxies such as benzocyclobutene (BCB) and SU-8 photoresists, which have an ultra-uniform polymer thickness and great bonding stability [16,17]. However, these types of polymers can only be dry etched by the fluorine etchants CF_4_ or SF_6_, which will also react with silicon, silicon nitride, and silicon oxide in the microstructure. Other types of adhesive polymer include negative photoresists (e.g., ULTRA-I 300), positive photoresists (e.g., AZ 5214), and polyimide (e.g., PI 2610), which can be easily removed by oxygen plasma isotropic etching. Nevertheless, the thermal and chemical stability of these kinds of polymer is weak [9,18]. Because of this, it is difficult to form the necessary interconnections through the intermediate polymer layer.

Poly (diallyl phthalate) (PDAP) is a novelty thermosetting polymer that was originally developed for nanoimprint processes [18,19]. Compared to the aforementioned photoresists and polyimides, it has better thermal and chemical stability, and it can be used to construct the interconnection routes. Moreover, different from BCB and SU-8, it can be removed by oxygen plasma without any other fluorine etchants.

In this work, a wafer-level 3D integration technology that uses PDAP as an intermediate bonding polymer was applied for integration with an SOI wafer and dummy CMOS wafer. Si–Glass adhesive bonding tests were performed to study the influences of different procedure parameters on adhesive bonding results. After that, integration tests were conducted to obtain the optimal 3D integration process conditions. Finally, 3D integration applications of system-on-chip were presented.

## 2. Materials and Experiment Methods

### 2.1. 3D Integration Materials

The MR-I 9000 series from the Micro Resist Technology (Berlin, Germany) was the available PDAP product that was commercially available. MR-I 9100M, MR-I 9150XP, and MA-N 1410 were used as the test adhesive bonding materials. MR-I 9100M was used as a standard nanoimprint resist, MR-I 9150XP was used as a customization nanoimprint resist, and MA-N 1410 was used as a standard negative photoresist. Micro Resist Technology (Berlin, Germany) supplied all of these polymers. MA-N 1410 was used to compare the PDAP-type polymers to one another in order to evaluate the bonding effect that is caused by different polymers. The difference between MR-I 9100M and MR-I 9150XP is their spin coating thicknesses under standard conditions (3000 rpm, 30 s). Table 1 shows the specifications for the spin coating and curing properties of the different polymers [20].

Different polymer thicknesses can be obtained by adjusting the spin speed during the process. The relationship between the polymer thickness and the spin coating speed can be described using the following equation [21]:

(1) $$t=\frac{kS^{2}}{\sqrt{RPM}}.$$

where *t* is the polymer thickness after the polymer has been spin coated, *k* is the proportionality constant of the polymer, *S* is the solute concentration of the polymer, and *RPM* is the spin speed. For an adhesive polymer, the different thicknesses at different spin speeds can be derived as:

(2) $$t_{1}=\frac{\sqrt{RPM_{0}}}{\sqrt{RPM_{1}}}t_{0}.$$

where *t*_1_ is the polymer thickness with spin speed *RPM*_1_, and *t*_0_ is the polymer thickness with the standard spin speed *RPM*_0_ (3000 rpm).

For the 3D integration tests, we used double-side polished silicon wafers with a diameter of 100 mm and a thickness of 475 µm, and these were integrated into the single-side polished SOI wafers, which had a diameter 100 mm and a thickness of 525 µm. The SOI wafers also comprised a 1500 nm thick SiO_2_ buried oxide layer and 600 nm thick monocrystalline Si SOI layer. The silicon wafers were used to fabricate the dummy CMOS wafers and had a topography of about 300 nm. This is similar to the topography of most foundry CMOS wafers. During the tests, the monocrystalline Si of SOI wafers were transferred and connected to the dummy CMOS wafers using the 3D-integration process. This verified the possibility of high-performance monocrystalline membrane application in CMOS-MEMS integration devices.

In addition, glass wafers with a diameter of 100 mm and a thickness of 300 µm were bonded to single-side polished Si wafers with a diameter of 100 mm and a thickness of 475 µm. This allowed any wafer bonding defects to be easily identified and characterized when observed through an optical microscope. All of the materials were commercially available.

### 2.2. 3D-Integration Procedure

**Figure 1:**
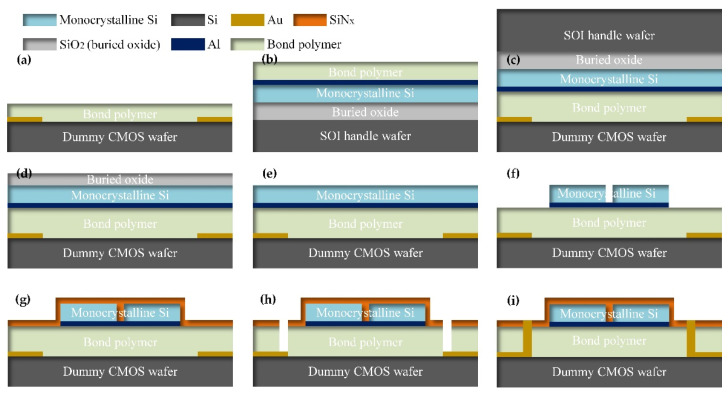
Three-dimensional integration with dummy CMOS wafer and SOI wafer: (**a**) fabrication of the dummy CMOS wafer and spin-coated adhesive polymer on it; (**b**) fabrication of the SOI wafer and spin-coated adhesive polymer on it; (**c**) adhesive bonding with dummy CMOS wafer and SOI wafer; (**d**) SOI wafer with the SI handle layer removed; (**e**) SOI wafer with etched buried oxide layer; (**f**) patterned the monocrystalline Si layer; (**g**) deposited SiN_x_; (**h**) Formed interconnection channels; (**i**) electroplating the interconnection Au pillars.

In the 3D integration test, the CB6L bonder and BA6 aligner (SUSS Micro-Tec, Garching, Germany) were used as the bonding equipment. The adhesive wafer bonding procedure consists of the following steps:

- First, clean the wafers in a standard acetone–isopropanol clean procedure (acetone ultrasonic cleaning 10 min, isopropanol ultrasonic cleaning 10 min, and deionized water rinse 2 min) and blow dry the wafers with N_2_. The wafers should then be baked in a vacuum oven at temperatures higher than 100 °C for 1 h in order to completely remove any remaining moisture.
- Second, the adhesive polymer is spin-coasted on the wafer surfaces in order for it to be bonded (as shown in Figure 1a,b). Then, the polymer-coated wafers are baked and pre-cured on a hot plate for a few minutes in order to remove the solvent in the polymer, making the polymer become partially crosslinked. In addition, oxygen plasma treatment is an option step that can be implemented after pre-curing to create a stronger bond.
- Third, the wafers are placed in a bonder fixture so that they can be manually aligned, a process that is conducted by clamping with a BA6 aligner. The pair of wafers are separated by three bonder fixture spacers. After that, the fixture with the wafer pairs is moved into the CB6L bonder chamber, which is then closed and sealed. The chamber is pumped to a pressure of less than 0.02 Pa, and this pressure is maintained for 5 min.
- Forth, the spacers should be removed, which can be achieved using the drive mechanism of the bonder, and the wafers will then be in contact with each other. Then, bonding pressure is applied to the backside of wafers by up-pressing chuck and down-pressing chuck. After that, the wafers are heated to the polymer-curing temperature with a temperature ramping speed of 5 °C/min, which is carried out using the hot plate within the up-pressing chuck and the down-pressing chuck. The curing temperature should be maintained for 40 min in order to ensure that the polymer is completely cross-linked. The temperature of the plate should then be decreased to 40 °C by blowing N_2_ with a temperature speed of about 5 °C/min.
- Finally, the bonder chamber is inflated to atmospheric pressure, and the bonding pressure is unloaded. The wafer pair should be removed from the chamber, and at this point, adhesive wafer bonding has been achieved (as shown in Figure 1c).

Before bonding, the Si wafer was patterned by lithography (MA6/BA6, SUSS Micro-Tec, Garching, Germany) and CF_4_-based reaction ion etch (RIE, Tegal 903e, Tegal, Petaluma, CA, USA) to make backside align marks. Then, Au/Ti layers with thicknesses of 270 nm/20 nm were deposited on the front side of the Si wafer via magnetron sputtering (FHR MS150 × 6 L, GCEMarket, Blackwood, NJ, USA). Additionally, the Au/Ti layers were patterned by lithography and Ar-Based ion beam etch (IBE, IBE-A-150, BCT, Beijing, China), in order to fabricate the dummy circuits (shown in Figure 1a). In addition, Al/Ti layers with thicknesses of 75 nm/20 nm were deposited onto the SOI wafer by means of magnetron sputtering (shown in Figure 1b).

After the adhesive bonding process was complete, the Si handle layer of the SOI wafer was removed by SF_6_-based inductive coupled plasma (ICP) etching (MPX HRM System, SPTS, Newport, UK), and the buried oxide layer was used as the etching stop layer during ICP etching (shown in Figure 1d). During Si etching, the SF_6_-based ICP etching process etched the SiO_2_ at a slow rate. Thus, the buried oxide layer should be thick enough to resist the ICP etching to remove the Si handle layer. The minimum thickness of the buried oxide *d*_lim_ can be approximately calculated as:

(3) $$d_{\lim}=\frac{D_{0}\Delta_{0}}{R_{0}}.$$

Here, *D*_0_ is the thickness of the Si handle layer in the SOI wafer, Δ_0_ is the etching inhomogeneity of the ICP equipment, and *R*_0_ is the etching selectivity ratio of Si/SiO_2_. The Si handle layer thickness of a commercially available SOI wafer with a 100 mm diameter is usually about 500 µm. The typical etching inhomogeneity of the MPX HRM system is ±5%, and the typical etching selectivity ratio of the ICP equipment is usually in the range of 20 to 35. As a result, the minor thickness of the buried oxide is about 1.43 µm to 2.5 µm. Chemical mechanical polishing (CMP, AP-380F, AM Technology, Ansan-si, South Korea) is used to homogenize the Si handle layer during ICP etching, which does not damage the SOI layer. The buried layer is removed by the buffered HF (H_2_O/HF = 10:1), and the etching was completely stopped at the SOI layer (shown in Figure 1e).

As shown in Figure 1f, the SOI layer was patterned by lithography and CF_4_-based RIE, and the Al circuit layer was etched by Ar-based ion beam etch (IBE, IBE-A-150, BCT, Beijing, China). An SiN_x_ layer that was 200 nm thick was deposited by plasma-enhanced vapor deposition (PECVD, Plasmalab System 100, OxFord Instrument, Abingdon, UK) and was used as the structural support layer for the 3D integration process (shown in Figure 1g). After that, the SiN_x_ layer was patterned by means of lithography and CF_4_-based RIE. On this basis, the polymer layer was anisotropically etched by the RIE (Plasmalab System 80, OxFord Instrument, Abingdon, UK), in which the SiN_x_ layer is used as etching mask. Various PDAP etching conditions were determined by the experiments (shown in Figure 1h). The metal pillars were constructed using electroplates to fill the etched holes (shown in Figure 1i). The magnitude of the electroplate current can be described as [22]:

(4) $$I_{e}=D_{e}S_{e}=\frac{60\gamma v}{100K\eta }S_{e}.$$

where *I_e_* is the magnitude of electroplating current, *D_e_* is the electroplating current density, *S_e_* is the area of the electroplate, *γ* is the density of the electroplate metal, *v* is the electroplate ratio, *K* is the electrochemical equivalent of the electroplate solutions, and *η* is the electroplating current efficiency. Table 2 shows the current calculation parameters for electroplating and the results of the gold and copper electroplating process.

## 3. Results and Discussion

### 3.1. Adhesive Wafer Bonding Results and Analysis

**Figure 2:**
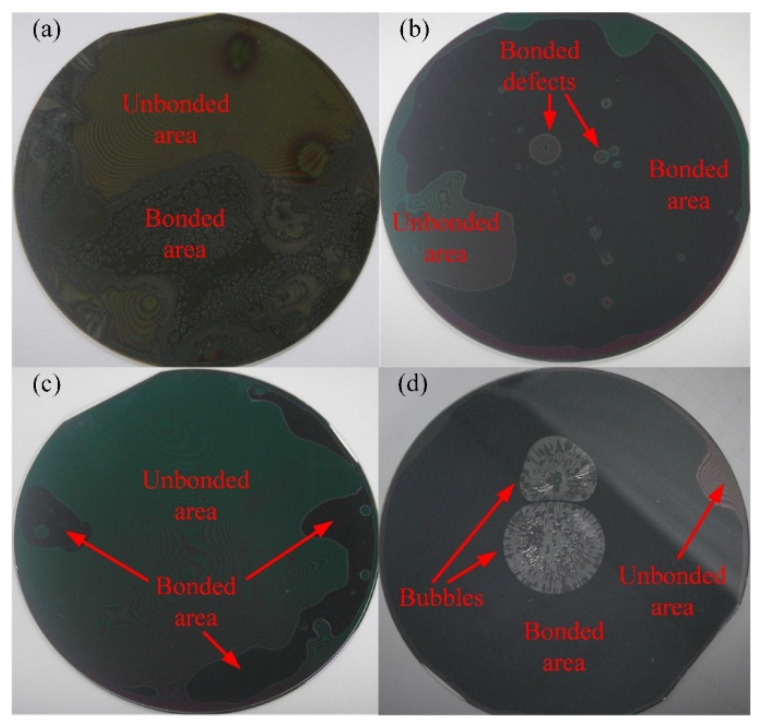
(**a**) Typical test result with MA-N 1410; (**b**) typical test result of PDAP with a low bonding pressure; (**c**) typical test result of PDAP with an excessive pre-curing temperature; (**d**) typical test result of PDAP with insufficient pre-curing.

The influences of process parameters on the bonding effects were analyzed by Si–Glass adhesive bonding tests. Adhesive bonding experiments are designed using the control variable method. Through these experiments, it was found that the type of polymer, bonding pressure, pre-curing condition, and spin coating condition have significant influence on the bonding results. The process parameters of serval typical tests are listed in Table 3, and the bonding results of these experiments are shown in Figure 2.

We performed three tests with MA-N 1410 as an adhesive polymer together with different process parameters. None of the test parameters that were set were able to achieve voidless bonding. After a typical bonding experiment using the same process parameters as those in test No.1 (Table 3), it was seen that the unbonded area accounted for more than half of the bonding interface (shown in Figure 2a). Moreover, many small voids were able to be observed over the entire unbonded area at the bond interface. This indicates that MA-N 1410 is not suitable for 3D integration.

Several MR-I 9100M and MR-I 9150XP tests were performed with different process parameters, with each process parameter being repeated twice. These experiment results indicate that PDAP-series polymers (MR-I 9100M, MR-I 9150XP, and so on) are appropriate for 3D integration and that these polymers have similar bonding properties. During these tests, it was determined that bonding pressure is the most important process parameters for polymer bonding. The unbonded area increased sharply when the bonding pressure decreased. Figure 2b shows a typical test result with a lower bonding pressure (1500 N), and the process parameters that were set for this test are listed in test No.2 (Table 3). The unbonded area and bonding defects can be reduced or even eliminated by significantly increasing the bonding pressure. Meanwhile, the bonding pressure should be adjusted along with the bonder limit and wafer strength.

The pre-curing condition for PDAP is another important process parameter that has an obvious influence on the bonding result. The pre-curing conditions for PDAP include pre-curing temperature and pre-curing time. The pre-curing temperature should be below the temperature at which the crosslinking reaction experiences a significant increase. Through the bonding tests with the process parameters from test No.3 (Table 3), it was found that adhesive bonding was hardly achieved (shown in Figure 2c). The excessive pre-curing caused a large unbonded area. On the other hand, insufficient pre-curing caused the generation of bubble defects at the bond interface (shown in Figure 2d). With the process parameters from test No.3 (Table 3), the solvent and the moisture in the polymer layer were not sufficiently removed by hotplate baking. A group of bubbles then formed at the bond interface, which was caused by the evaporation of the residual solvent and moisture.

**Figure 3:**
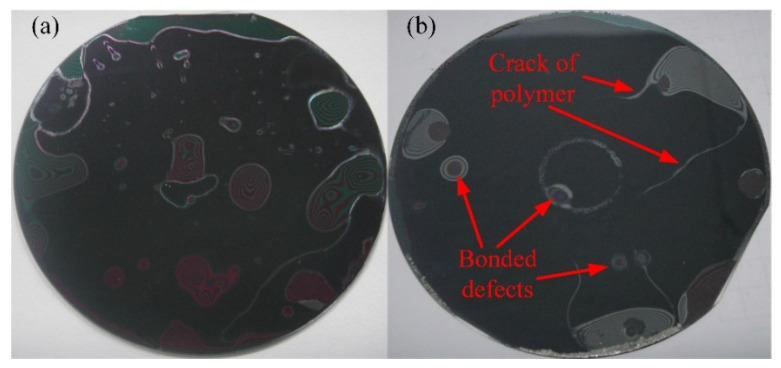
(**a**) Typical test result with storage after pre-curing and using unclean wafers; (**b**) typical test result with storage after pre-curing.

Furthermore, it was found that cleanliness and immediacy have a certain effect on the bonding results. A bonding experiment was conducted using the process parameters from test No.5 (Table 3) and using unclean wafers, meaning that the wafers were stored in the N_2_ tank for 2 days after the polymers had been pre-cured. By the time that the test took place, it could be observed that there were many cracks in polymer layer and that there were various particle defects at the bonding interface (shown in Figure 3a). Another test using the process parameter from No.6 (Table 3) and using the wafers that had been stored in the N_2_ tank for 2 days was conducted. During this test, many cracks were still found in the polymer layer, and it was determined that the polymer pre-curing process had been insufficient (shown in Figure 3b). Moreover, the bonding defects that were seen in the particles were decreased by cleaning the bonding wafers and by increasing the thickness of the polymer layer.

**Figure 4:**
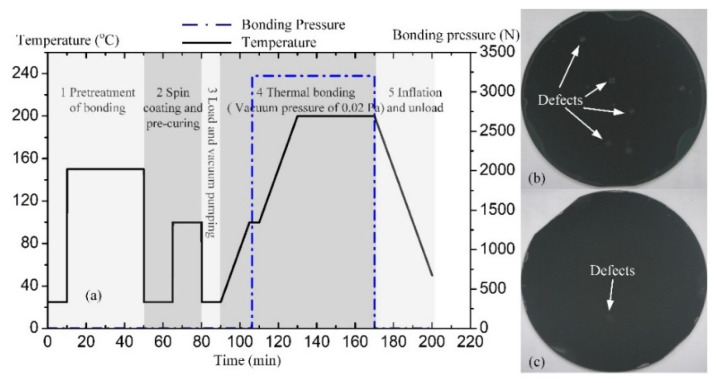
(**a**) Technological process curve of the PDAP adhesive bonding; (**b**) typical test result of MR-I 9100M with the optimal process parameters; (**c**) typical test result of MR-I 9150XP with the optimal process parameters.

Through these experiments, we were able to achieve the optimal parameters for PDAP adhesive, and the technological process curve is shown in Figure 4a. After two bonding tests with the process parameters from test No.7 and No.8 (Table 3), it was seen that the voidless PDAP adhesive bonding is achieved (shown in Figure 4b,c).

MR-I 9100M and MR-I 9150XP both belong to the PDAP series of polymers, which is commercially available as nanoimprint resist. During the bonding process, these polymers demonstrate similar process properties, with the exception of the polymer thickness in the standard spin coating condition. As shown in Figure 4b, it was seen that the bond interface has several obvious particle defects (with the MR-I 9100M). By comparison, it is quite rare to find the particle defects in the bond interface (shown in Figure 4c). The polymer thickness of MR-I 9100M is about 1000 nm in standard spin coating conditions, and the polymer thickness of MR-I 9150XP is about 1500 nm in the same conditions. Particle defects can be reduced or eliminated by increasing the thick-ness of the polymer layer. On the other hand, if the polymer layer is excessively thick, then it will cause the difficulties with the 3D interconnection. The thickness of the polymer layer should be adjusted via lab cleanliness and by adjusting the 3D integra-tion requirements.

**Figure 5:**
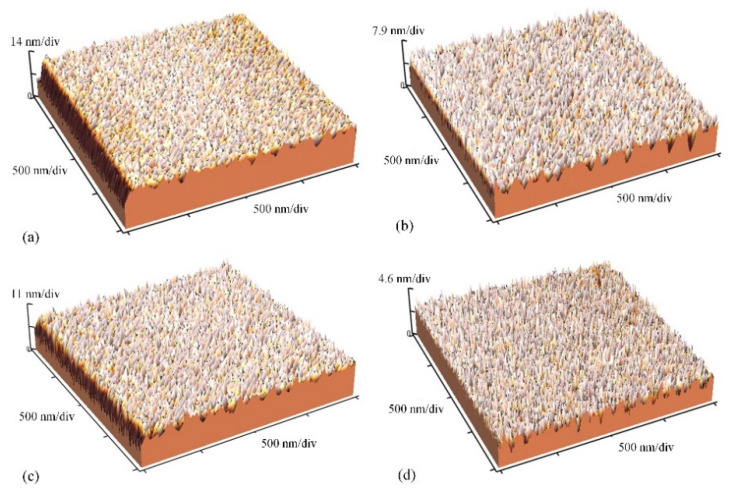
(**a**) AFM test results of MR-I 9100M without O_2_ plasma treatment; (**b**) AFM test results of MR-I 9100M with O_2_ plasma treatment; (**c**) AFM test results of MR-I 9150XP without O_2_ plasma treatment; (**d**) AFM test results of MR-I 9150XP with O_2_ plasma treatment.

In order to compare MR-I 9100M and MR-I 9150XP, four experiments were conducted using the optimal process parameters that can be seen in Figure 4a. Both the MR-I 9150XP were spin coated in standard conditions (3000 rpm, 30 s), and the thickness of the polymer layers was about 1500 nm. According to Equation (2), both of the MR-I 9100Ms were coated at the spin speed of 1330 rpm, and the thicknesses of the polymers were similar to those that were used during MR-I 9150XP coating. Among these tests, the O_2_ plasma treatment was used in one test with MR-I 9100M and in one with MR-I 9150XP. As shown in Figure 5, the roughness measurement was conducted with an atomic force microscope (AFM, Veeco M5, Plainview, NY, USA). Table 4 lists the AFM test results of the 1 × 1 µm^2^ samples in the middle of the test area and include the average roughness *R_a_*, maximum roughness *R_z_*, average maximum roughness *R_t_*, and root mean square of roughness *R_q_*. According to these results, it can be determined that the surface roughness of the polymer is smoother when the standard spin coating conditions are used. When non-standard conditions are used, then surface roughness of the polymer is slightly rougher than it is when standard conditions are used. In addition, the topography of polymer was decreased after the O_2_ plasma treatment. When the bond interface has a smooth surface, it is easier to obtain better bond results. 

### 3.2. 3D Integration Results and Applications

**Figure 6:**
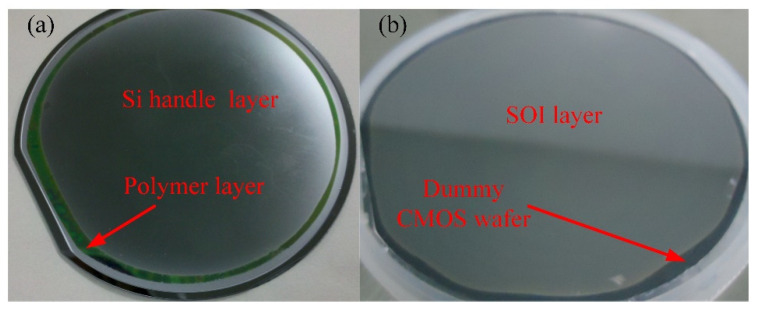
(**a**) The Si handle layer etching test result without CMP; (**b**) the SOI layer transfer test result with CMP.

To demonstrate the suitability of PDAP as an intermediate layer for 3D integration, the SOI layers were transferred from the SOI wafers (handle wafers) to dummy CMOS wafers. After adhesive bonding with the optimal process parameters, the Si handle layer of the SOI wafer was removed by the ICP etching process, in which the bulk etching velocity ranged from 4.7 to 5.2 µm/min. During the ICP etching processes, it is recommended that 30 min be added when the process is halfway through. Figure 6a shows the results of Si layer etching when CMP was not used. The edges of the wafer were etched to intermediate the polymer layer, where the center of the wafer still had a thick Si handle layer. The non-uniformity accumulation of ICP etching caused this result. The non-uniformity accumulation can be approximately calculated by Equation (3). It can be solved by increasing the thickness of the buried oxide layer or with the addition of a CMP procedure. After the ICP etching procedure, the buried oxide layer can be etched by the buffered HF. When the surface of the wafer was hydrophobic, the buried oxide layer was completely removed, and the SOI layer was transferred from the SOI wafer to the dummy CMOS wafer. Figure 6b shows the final transfer test result achieved by ICP etching over 102 min, at a CMP of 30 min, and after buffered HF etching for 11 min.

**Figure 7:**
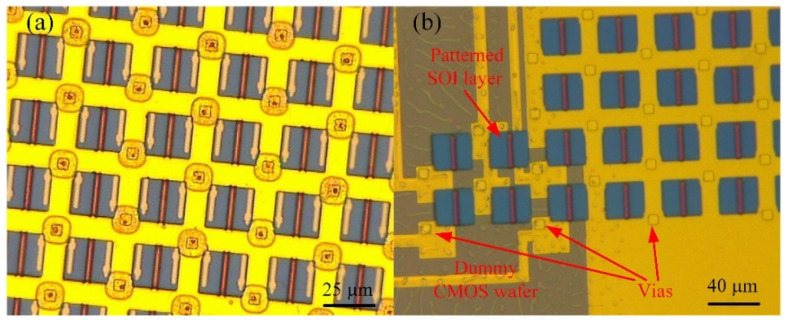
(**a**) The PDAP bowing etching result with the sidewall of vias; (**b**) the PDAP etching result with the interconnection vias of regular shape.

The SOI layer and Al circuit layer were patterned using the lithography, RIE, and IBE procedures. Then, a functional SiN_x_ layer of 150 nm was deposited by PECVD and was patterned by RIE. The polymer layer was anisotropically etched using the SiNx layer as an etching mask, which was used to form the interconnection routes. The PDAP polymer layer was able to be etched by O_2_-based RIE easily, creating serious bowing etching along the sidewalls of the interconnection routes (shown in Figure 7a). This will cause the 3D integration of the interconnection process to short circuit, resulting in 3D integration failure. During the experiments, multiple etching procedures were testing. Through the experiments, the background vacuum degree, reaction gas ration (O_2_), assistant gas ration (Ar), and reaction pressure were found to have a significant influence on the PDAP etching results. Two suggested PDAP etching conditions and the etch rates of each condition are listed in Table 5. Using both of the process conditions from Table 5, regularly shaped interconnection routes were obtained. Figure 7b shows a PDAP etching result with regularly shaped interconnection routes that were attained according to the procedure conditions from No.1 (Table 5). 

**Figure 8:**
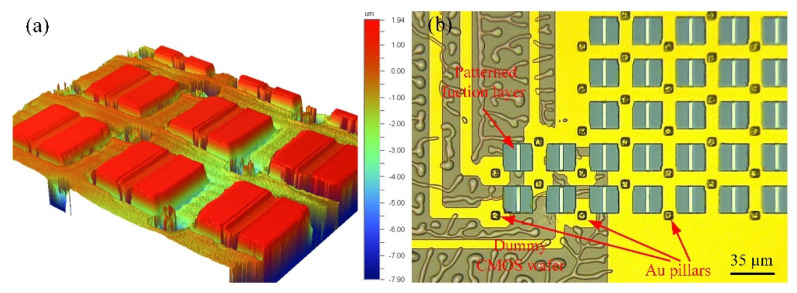
(**a**) The topography measurement results of the wafer with the profilometer; (**b**) the Au electroplating results.

Finally, the interconnection routes are filled with the electroplate metal, and 3D integration with monocrystalline Si and a dummy CMOS wafer is achieved. The electrical pillars that travel through the interconnection routes can be constructed by electroplating copper, gold, and nickel. Considering the influence of oxidation and surface roughness, electroplating with gold (Neutronex 309, Enthone, Bridgeview, IL, USA) was used in the tests that were conducted for this study. After Au electroplating, topography measurements of the wafer were conducted through the use of a profilometer (Wyko NT1100, Vecco, Plainview, NY, USA); it was seen that the interconnection of the Au pillar increased without over electroplating (shown in Figure 8a). With the micrograph, it can be seen that the shape of the Au pillars is regular. Independent interconnections between the dummy CMOS wafer and SOI layer are established.

**Figure 9:**
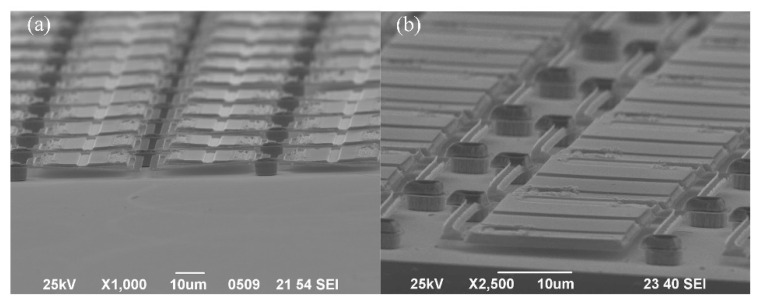
(**a**) The 3D integration application of micro-bolometer array; (**b**) The 3D integration application of micro-bridge resistor array.

After 3D integration, the bonding polymer layer can be sacrificially removed by O_2_ plasma isotropy dry etching in order to construct suspended microbridge structures. Figure 9a shows a 320 × 240 micro-bolometer array for infrared thermal imaging, which was fabricated based on 3D integration with SiGe/Si MQWs materials and dummy CMOS wafers. Figure 9b shows a 120 × 80 micro-bridge resistor array that can be used to generate an infrared scene fabricated based on 3D integration with monocrystalline silicon and dummy CMOS wafers. This demonstrates that 3D integration based on PDAP adhesive bonding is suitable for the fabrication of system-on-chip that enables integration with MEMS and ICs.

## 4. Conclusions

Wafer-level 3D integration technology using PDAP as an intermediate bonding polymer was effectively applied for integration with an SOI wafer and a dummy CMOS wafer. The influences of the procedure parameters on the adhesive bonding effects were determined by Si–Glass adhesive bonding tests. In these experiments, it was found that bonding pressure, pre-curing conditions, spin coating conditions, and cleanliness have a significant influence on the bonding results. The optimal procedure parameters of the PDAP adhesive bonding were obtained through analysis and comparison. According to this, the 3D integration tests were carried out. During the tests, process optimization focused on Si handle layer etching, PDAP layer etching, and Au pillar electroplating. The optimal process conditions for 3D integration process were achieved. Three-dimensional integration applications for the micro-bolometer array and micro-bridge resistor array were presented. Three-dimensional integration based on PDAP adhesive bonding provides a promising total solution for the fabrication of system-on-chip by MEMS and ICs integration, especially for the fabrication of low-cost suspended microstructures on-CMOS-chip systems. 

## Figures and Tables

**Table 1:** The spin coating, curing, and thermal stability parameters for the tests.

Material	Curing Temperature (°C)	Thickness @ 3000 rpm	Thermal Stability (°C)
MR-I 9100M	150–225	1000 nm	260
MR-I 9150XP	150–225	1500 nm	260
MA-N 1410	100–120	1000 nm	160

**Table 2:** The electroplating current calculation results of the gold and copper electroplating.

Metal	*γ* (g/cm^3^)	*v* (nm/min)	*K* (g/Ah)	*η*	*D_e_* (A/dm^2^)	*S_e_* (dm^2^)	*I* (mA)
Au	19.3	150	7.349	0.95	0.249	0.04	10
Cu	8.93	100	1.186	0.95	0.476	0.04	19

**Table 3:** The process parameters of typical bonding tests.

No	Label	RPM	Pre-Curing Temperature (°C)	Pre-Curing Time (min)	Bonding Pressure (N)	Bonding Temperature (°C)
1	MA-N 1410	3000	100	3	1500	120
2	MR-I 9100M	1330	100	15	1500	200
3	MR-I 9150XP	3000	150	15	1500	200
4	MR-I 9100M	1330	100	5	3200	200
5	MR-I 9100M ^1^	3000	100	10	3200	200
6	MR-I 9150XP ^2^	3000	100	5	3200	200
7	MR-I 9100M	3000	100	15	3200	200
8	MR-I 9150XP	3000	100	15	3200	200

^1^ In this test, the wafers were not cleaned. After pre-curing, the wafers were stored in a N_2_ tank for 2 days. ^2^ After pre-curing, the wafers were stored in a N_2_ tank for 2 days.

**Table 4:** The AFM test results.

Label	*R_a_* (nm)	*R_z_* (nm)	*R_t_* (nm)	*R_q_* (nm)
Figure 5a	1.85	27.43	20.27	2.75
Figure 5b	1.33	13.55	8.96	1.66
Figure 5c	1.73	21.13	13.42	2.25
Figure 5d	0.86	9.28	4.33	1.08

**Table 5:** The suggested conditions and etch rates of PDAP etching.

No	Background Vacuum Degree	RF Power	Reaction Gas Ration (sccm)	Assistant Gas Ration (sccm)	Reaction Pressure	Etch Rate (nm/min)
1	0.002 Pa	200 W	40	0	2.66 Pa	410
2	0.005 Pa	200 W	40	10	3.99 Pa	580

## References

[1] Ko C.T., Chen K.N. (2012). Low temperature bonding technology for 3D integration. Microelectron. Reliab..

[2] Jiang J., Parto K., Cao W., Banerjee K. (2019). Ultimate monolithic-3D integration with 2D materials: Rationale, prospects, and challenges. IEEE J. Electron. Devices Soc..

[3] Koester S.J., Young A.M., Yu R.R., Purushothaman S., Chen K.N., La Tulipe D.C., Rana N., Shi L., Wordeman M.R., Sprogis E.J. (2008). Wafer-level 3D integration technology. IBM J. Res. Dev..

[4] Koyanagi M. (2015). Recent progress in 3D integration technology. IEICE Electron. Express.

[5] Reda S. (2017). 3D integration advances computing. Nature.

[6] Kim S.E., Kim S. (2015). Wafer level Cu–Cu direct bonding for 3D integration. Microelectron. Eng..

[7] Higurashi E., Suga T. (2016). Review of low-temperature bonding technologies and their application in optoelectronic devices. Electron. Commun. Jpn..

[8] Higurashi E. (2013). Low-temperature bonding technologies for photonics applications. ECS Trans..

[9] Niklaus F., Stemme G., Lu J.Q., Gutmann R.J. (2006). Adhesive wafer bonding. J. Appl. Phys..

[10] Niklaus F., Kumar R.J., McMahon J.J., Yu J., Lu J.Q., Cale T.S., Gutmann R.J. (2006). Adhesive wafer bonding using partially cured benzocyclobutene for three-dimensional integration. J. Electrochem. Soc..

[11] Forsberg F., Lapadatu A., Kittilsland G., Martinsen S., Roxhed N., Fischer A.C., Stemme G., Samel B., Ericsson P., Høivik N. (2015). CMOS-integrated Si/SiGe quantum-well infrared microbolometer focal plane arrays manufactured with very large-scale heterogeneous 3-D integration. IEEE J. Sel. Top. Quantum Electron..

[12] Niklaus F., Kälvesten E., Stemme G. (2001). Wafer-level membrane transfer bonding of polycrystalline silicon bolometers for use in infrared focal plane arrays. J. Micromech. Microeng..

[13] Zimmer F., Lapisa M., Bakke T., Bring M., Stemme G., Niklaus F. (2011). One-megapixel monocrystalline-silicon micromirror array on CMOS driving electronics manufactured with very large-scale heterogeneous integration. J. Microelectromech. Syst..

[14] Schmidt J.U., Friedrichs M., Bakke T., Voelker B., Rudloff D., Lakner H. Technology development for micromirror arrays with high optical fill factor and stable analogue deflection integrated on CMOS substrates. Proceedings of the MEMS, MOEMS, and Micromachining III, International Society for Optics and Photonics.

[15] Guerre R., Drechsler U., Bhattacharyya D., Rantakari P., Stutz R., Wright R.V., Milosavljevic Z.D., Vaha H.T., Kirby P.B., Despont M. (2010). Wafer-level transfer technologies for PZT-based RF MEMS switches. J. Microelectromech. Syst..

[16] Makihata M., Tanaka S., Muroyama M., Matsuzaki S., Yamada H., Nakayama T., Yamaguchi U., Mima K., Nonomura Y., Esashi M. (2011). Adhesive wafer bonding using a molded thick benzocyclobutene layer for wafer-level integration of MEMS and LSI. J. Micromech. Microeng..

[17] Agirregabiria M., Blanco F.J., Berganzo J., Arroyo M.T., Fullaondo A., Mayora K., Ruano-Lopez J.M. (2005). Fabrication of SU-8 multilayer microstructures based on successive CMOS compatible adhesive bonding and releasing steps. Lab Chip.

[18] Bleiker S.J., Dubois V., Schröder S., Stemme G., Niklaus F. (2017). Adhesive wafer bonding with ultra-thin intermediate polymer layers. Sens. Actuators A Phys..

[19] Niklaus F., Decharat A., Forsberg F., Roxhed N., Lapisa M., Populin M., Stemme G. (2009). Wafer bonding with nano-imprint resists as sacrificial adhesive for fabrication of silicon-on-integrated-circuit (SOIC) wafers in 3D integration of MEMS and ICs. Sens. Actuators A Phys..

[20] Micro Resist Technology. https://www.microresist.de/.

[21] Zhong F., Dong T., Yong H., Yan S., Wang K. (2013). Void-free wafer-level adhesive bonding utilizing modified poly (diallyl phthalate). J. Micromech. Microeng..

[22] Giurlani W., Zangari G., Gambinossi F., Passaponti M., Salvietti E., Di Benedetto F., Caporali S., Innocenti M. (2018). Electroplating for decorative applications: Recent trends in research and development. Coatings.

